# A novel rind puncture technique to measure rind thickness and diameter in plant stalks

**DOI:** 10.1186/s13007-020-00587-4

**Published:** 2020-04-01

**Authors:** Will H. Seegmiller, Jadzia Graves, Daniel J. Robertson

**Affiliations:** Department of Mechanical Engineering, University of Idaho, 875 Perimeter Dr., MS 0902, Moscow, ID 83844 USA

**Keywords:** Rind, Thickness, Diameter, Penetration, Puncture, Stalk, Lodging, Plant, High throughput, Phenotype, Strength

## Abstract

**Background:**

Measurements of rind and culm thickness and stem radius/diameter are important to biomechanical, ecological and physiological plant studies. However, many methods of measuring rind thickness and diameter are labor intensive and induce plant fatality. A novel rind puncture methodology for obtaining measurements of rind thickness and diameter has been developed. The suitability of the new method for implementation in plant studies is presented.

**Results:**

The novel rind puncture technique was used to obtain measurements of rind thickness and diameter for samples of Poison Hemlock (*Conium maculatum*). The rind puncture measurements were strongly correlated with caliper measurements (R^2^ > 0.97) and photographic image analysis measurements (R^2^ > 0.84). The capacity for high throughput measurements using the rind puncture technique was determined to exceed that of caliper measurements and image analysis techniques.

**Conclusions:**

The rind puncture technique shows promise as a high throughput method for determining rind thickness and diameter as it is cost effective and non-lethal. The authors are currently working to develop a custom handheld apparatus to allow the novel rind puncture method to be used in field work. High throughput field-based measurements of rind thickness and diameter are needed to help address the problem of stalk lodging (failure of grain crops to remain upright until harvest).

## Background

Measurements of stem diameter and rind thickness are important to physiological, biomechanical and ecological plant studies [1–6]. However, these measurements are often labor intensive, low throughput, and/or require expensive imaging equipment [7]. Measurements of rind thickness typically require either expensive biomedical imaging procedures (e.g., X-ray computed tomography) or destructive sectioning procedures that result in plant fatality (i.e. using manual or powered cutting tools) [4, 8, 9]. As an alternative to these procedures, the authors developed a novel, minimally invasive rind puncture test methodology to measure rind thickness and diameter that does not induce plant fatality. The authors hypothesized that the new procedure would enable high throughput rates while maintaining measurement accuracy.

Commonly used tools to measure rind thickness and diameter in previous studies include calipers [10–12], photographic image analysis [13] and X-ray computed tomography [14]. Several methods for indirectly predicting rind thickness have also been presented. For example, correlations have been established in sorghum (*Sorghum bicolor*) relating weight and circumference to rind thickness, thereby enabling an indirect estimation of rind thickness based on measurements of weight and circumference [15]. However, caliper measurements, image analysis, and the weight/circumference methods all require destructive and labor-intensive sectioning processes that induce plant fatality. X-ray computed tomography is capable of generating accurate measurements without inducing permanent damage to the plant but has the disadvantage of being impractical for field-based measurements. In addition, computed tomography requires acquisition of expensive imaging equipment and software and is fairly time intensive.

To evaluate the utility of the novel rind puncture test methodology to measure rind thickness and diameter of plant stems it was directly compared to two other common measurement techniques. In particular, measurements of rind thickness and diameter were acquired using calipers, photographic image analysis, and the novel rind puncture test methodology. The time to complete each measurement technique along with its associated cost and accuracy were compared.

## Results

A total of 113 poison hemlock specimens were included in the study. However, 17 fractured while trying to extract cross-sectional samples for image analysis. Image analysis results are therefore presented for 96 samples, whereas results for the puncture and caliper measurements include all 113 samples. Summary statistics including the mean, range, standard deviation and variance for measurements of diameter and rind thickness are presented in Table 1.

**Table 1:** Summary of statistical features of each measurement data set

	Diameter measurements (mm)			Rind thickness measurements (mm)		
	Caliper	Image analysis	Puncture method	Caliper	Image analysis	Puncture method
Mean	13.78	13.89	14.05	2.37	2.39	2.61
Range	16.84	15.96	16.42	2.76	2.76	2.97
Standard deviation	3.17	3.096	3.71	0.6	0.62	0.71
Variance	10.03	9.59	13.75	0.35	0.39	0.51

Two researchers measured each plant sample using the same set of digital calipers. The same two researchers also performed image analysis on each sample. The interuser variabilities between researchers for the diameter measurements with calipers and image analysis were 1.50% and 1.64%, respectively. For the rind thickness measurements with the same tools, the interuser variabilities were 7.71% and 8.68%, respectively. As the rind puncture tests were machine actuated no interuser variability data was available for the puncture test method.

### Time required to measure samples

The time required to perform each measurement was recorded to enable comparison of each methods potential for high throughput phenotyping. Table 2 shows a comparison of the time required to prepare samples for measurement and the time required to carry out each step of the measurement.

**Table 2:** Comparison of time required to take measurements of diameter and rind thickness with each method

Calipers	
Measure diameter	150 min
Cut internode	150 min
Measure rind thickness	120 min
Record measurements	10 min
Total	430 min
Image analysis	
Cut cross sections	450 min
Scan cross sections	30 min
Load images into program	20 min
Calculate rind thickness and diameter	120 min
Record measurements	10 min
Total	630 min
Rind puncture method	
Puncture stalks	60 min
Data analysis to calculate rind thickness and diameter	5 min
Total	65 min

The time reported is the time to complete the measurements for all 113 samples in the study

### Agreement between methods

Linear correlation analysis was employed to compare the results of the three measurement techniques employed in this study (i.e., image analysis, caliper measurements and the novel rind puncture technique). Table 3 shows the coefficients of correlation between each method. The average diameter and rind thickness values from the two researchers who took image analysis and caliper measurements were used to compute the coefficients of correlation presented Table 3. All methods showed strong agreement, indicating the ability of the rind puncture method to obtain accurate measurements of rind thickness and diameter.

**Table 3:** Comparison of R squared values between each measurement method

	Caliper	Image analysis
R^2^ values between methods—diameter		
Puncture method	0.9939	0.9700
R^2^ values between methods—rind thickness		
Puncture method	0.8623	0.8410

## Discussion

Each method of measurement investigated in the current work (including the novel rind penetration method) had its attendant advantages and disadvantages. Comparisons can be drawn between the different measurement techniques in the following areas: cost of tools required, training required to perform the measurements, time required to carry out the measurements, the random error introduced by the user, and consistency of measurement values compared to the other methods studied in this paper. Each of these areas is discussed in the paragraphs below.

### Cost of tools

Each measurement method used in this study required at least one tool. The caliper method required a pair of digital calipers. A representative cost of a pair of digital calipers is $20 to $100. The image analysis technique required a computer, a scanner, and software. The cost of the computer and scanner together are estimated at $400 to $1000. The software (i.e. ImageJ) was open source and therefore incurred no cost. The rind puncture method required an Instron universal testing frame, a computer, and a MATLAB license. Together, these items cost approximately $50,000. In summary, the most expensive method was the rind puncture method by a wide margin, while the least expensive was the caliper method.

### Training required

Training for all three methods took approximately the same amount to time to carry out. For the caliper method, a 10 min demonstration of proper caliper usage was all that was required. For the image analysis method, researchers watched a 10 min video to familiarize themselves with the software tools they would be using. For the rind puncture method, training consisted of a 10 min demonstration of the procedure. In other words, each method required approximately 10 min of training.

### Time to complete measurements

The total time spent by all researchers in carrying out the various measurements are summarized in Table 3. The image analysis method was the most time consuming, requiring 630 min to complete. The most time intensive process for image analysis was sectioning the stalk samples (450 min). The caliper method was the next most time intensive requiring 430 min to complete. The least time intensive method was the rind puncture method, which required only 65 min. It should be noted that several automated image analysis algorithms have been presented in the literature that could reduce the time to scan and compute stalk diameter and rind thickness [5, 6, 16–21]. However, the authors are unaware of any reported high throughput sectioning procedures that would reduce time to section stalk cross-sections below the reported 450 min required in this study. Thus, the rind puncture methods would still be significantly faster even if automated image analysis algorithms were employed.

### Interuser variability

Inter user variability was measured for the caliper and image analysis methods and was found to be small to moderate (< 2% for diameter measurements and < 9% for rind thickness measurements). Because a given measurement site can only be punctured a single time, no comparisons of inter user variability were made for the puncture method. The authors expect inter user variability for rind thickness measurements to be significantly higher when measuring pith filled plant stems as it can be difficulty to determine the boundary between pith and rind.

### Agreement between methods

To determine the level of agreement between measurement systems a linear correlation analysis was conducted. Each system exhibited coefficients of determination (R^2^ values) greater than 0.84 (see Table 3). The high level of agreement between the different methods suggests that any of these methods could be used to obtain accurate measurements of rind thickness and diameter. The attendant advantages and disadvantages of each method are discussed in the sections below.

### Advantages/disadvantages of caliper measurements

Calipers are an inexpensive tool. They are easy to obtain and easy to use. They can be used with equal ease in a laboratory or in the field. However, their capacity for high throughput measurements of rind thickness is limited, making measurements of large sample sets impractical. Calipers would be a preferred tool in studies requiring immediate measurements of rind thickness of plant stalks/stems for a relatively small sample set (i.e., < 100 samples/user).

### Advantages/disadvantages of image analysis methods

The Image analysis method to determine stalk/stem diameter and rind thickness was effective but required more sample preparation (i.e. cutting a thin cross section capable of being placed on a flatbed scanner) than the other two methods. In this study 15% of the samples (i.e. 17 internodes) were destroyed during sectioning. This method requires tools that are not easily portable to the field, limiting this method primarily to laboratory settings. Image analysis would be a preferred method for experiments requiring measurements of rind thickness of small to large sample sizes in laboratory settings, so long as the samples are easy to section. An added advantage of the image analysis method is that it does not requiring contacting the sample. When measuring soft or deformable samples caliper readings are highly dependent upon the amount of force the user applies to the sample. Image analysis techniques and other non-contact methods are often more suited to measure such samples as compared to calipers.

### Advantages/disadvantages of the rind puncture method

The rind puncture method is the only non-lethal method of measuring rind thickness that could potentially be used in a field setting. For example, puncture tests are frequently used in field studies of maize (*Zea mays*) and other grasses to assess stalk strength without inducing plant fatality (e.g., 18). In this study a universal material testing frame/system was used to conduct the puncture tests. Materials testing frames are largely immobile and inappropriate for field work. The authors chose to use a universal testing frame to validate the puncture test methodology. However, they are currently developing a portable handheld device to conduct puncture tests of plant stems and stalks. The primary advantage of such a device would be the ability to determine stalk diameter and rind thickness in the field without inducing plant fatality. The authors are not aware of any other methods to non-destructively measure rind thickness in a field. In the meantime, laboratory-based puncture tests which utilize a universal testing system can offer a high degree of automation, allowing for high throughput measurements of rind thickness and diameter. Such tests are best suited for large sample sets (> 100 samples). Table 4 presents a quantitative summary of each methods advantages and disadvantages. The last column of the table presents the average of the R^2^ values between the given technique and the two other methods investigated in the study.

**Table 4:** Comparison of possible advantages and disadvantages for each measurement method

	Equipment cost	Training time (min)	Time to measure 113 samples (min)	Inter user variability	Average R^2^ (diameter)	Average R^2^ (rind thickness)
Caliper	~ $50	10	430	1.5% and 7.71%	0.9854	0.8854
Image analysis	~ $500	10	630	1.64 and 8.68%	0.9735	0.8748
Puncture method	~ $50,000	10	65	Not studied. Assumed negligible	0.9820	0.8517

### Complexity of obtaining accurate rind thickness measurements

Rind thickness measurements for all methods demonstrated lower R^2^ values than diameter measurements. This was partly due to difficulty associated with identifying the correct plane of measurement. For example, for the image analysis and caliper measurements there was uncertainty as to what two points should be used to calculate rind thickness when a geometric irregularity in the stalk cross-section was at or near the measurement location. Caliper measurements of rind thickness were also sensitive to variations in pressure applied by the user.

High throughput measurement techniques are needed to advance many fields of plant science. The lack of high throughput phenotyping methods is a major bottleneck to plant improvement and to increasing food security worldwide [22]. For example, high throughput methods of measuring rind thickness and diameter will greatly aid in addressing the problem of stalk lodging (plants breaking prior to harvest). Stalk lodging is a multi-billion dollar a year problem in grain crops and causes harvest losses that are estimated to range from 5 to 20% globally [23]. Stalk geometry (diameter and rind thickness) account for as much as 70% of the variation in stalk strength [6]. Because most plant stems are tapered measurements of diameter and rind thickness need to be acquired at multiple locations along the length of the stem. For example, the novel puncture technique outlined in this paper was recently used by the authors to acquire diameter and rind thickness measurements of every internode of a set of 1000 maize stalks. The collected data enabled an in-depth analysis of the taper and load bearing structure of the measured stalks and revealed insights into the structural efficiency of maize stems [24].

## Conclusions

The rind puncture technique described in this paper is a viable method to obtain measurements of rind thickness and diameter. The method is non-lethal, easy to perform, and has high throughput. It is recommended for use in studies with large sample sets. The authors are currently working to develop a custom handheld apparatus to allow the novel rind puncture method to be used in field work.

## Methods

### Plant materials

Poison hemlock samples were harvested on the morning of June 22, 2018 in Whitman County, Washington. Plants were at flowering stage, without any visible signs of disease. Each stalk was cut through the first above ground internode and through the first internode exhibiting a diameter less than 7 mm (i.e., all internode samples included in the study had a diameter larger than 7 mm). A total of 25 plants were harvested, resulting in 113 internodes total included in the study. Prior to taking any measurements each internode sample was marked with a permanent marker to indicate the locations (apical to basal) at which diameter and rind thickness measurements would be taken. All measurements and sample preparations (presented below) were accomplished within 24 h of harvesting. All measurements of stalk diameter presented in this work refer to measurements of the stalk’s minor diameter (i.e., the minimum diameter of the stalk cross-section).

Each of the 113 internodes described above was measured using three techniques (calipers, image analysis, and the novel rind puncture technique). The techniques are presented below in the same chronological order in which the experiments were conducted.

### Caliper measurements of diameter

Diameter measurements were acquired using a pair of digital calipers. Diameter measurements were acquired by placing the jaws of the calipers around the stalk and repeatedly rotating the stalk to identify and mark the orientation of the minimum reading (i.e., minor diameter of the cross-section). All results were recorded on an electronic spreadsheet. Two technicians measured each internode using the same set of calipers.

### Novel puncture method of measuring rind thickness and diameter

**Fig. 1:**
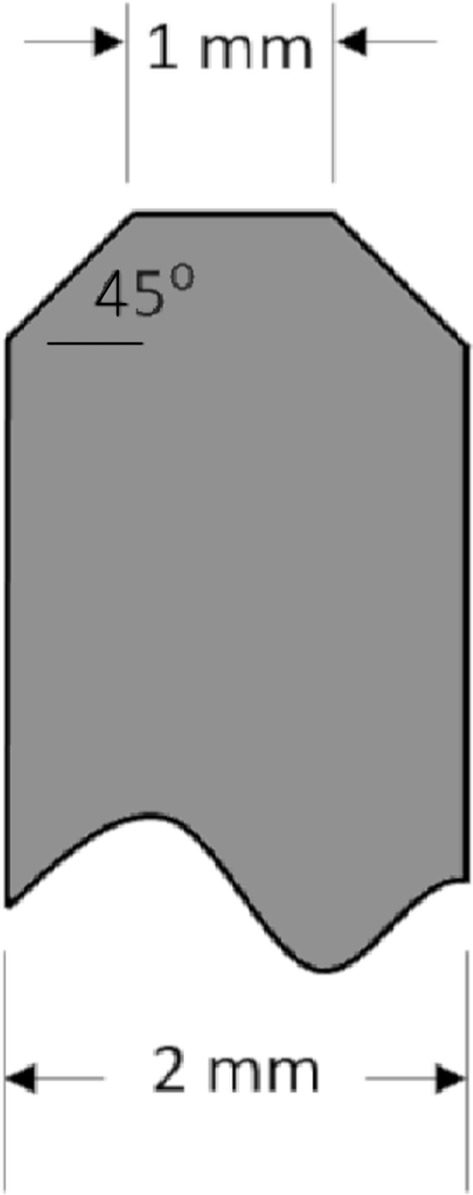
Diagram of probe geometry

**Fig. 2:**
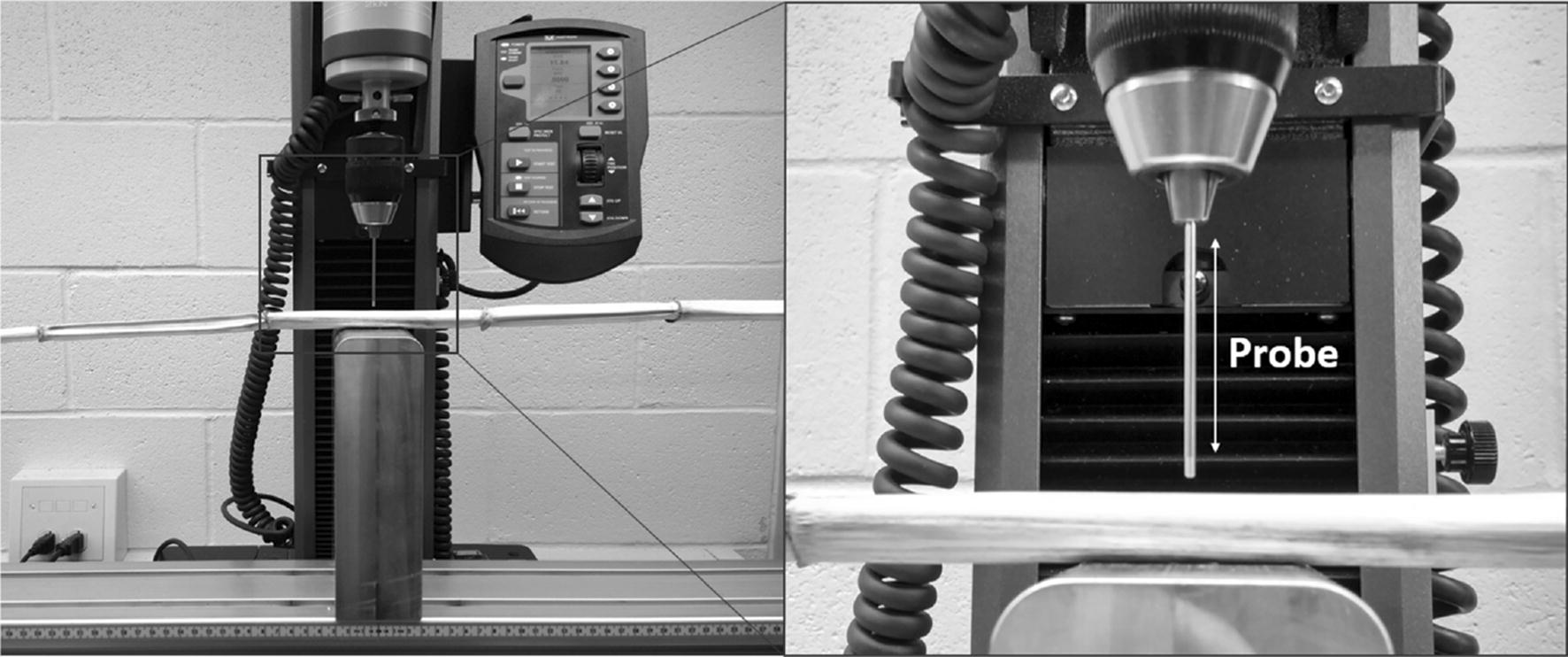
Puncture test being performed on a Poison Hemlock stalk. The block supporting the plant sample has a 5 mm diameter, 10 mm deep hole in it to allow the probe to puncture through the entire stalk without hitting the support block

A Universal Testing System (Instron, model # 5944, Norwood MA) was used to puncture the center of each internode sample in the direction of the minor cross-sectional axis (i.e., in the direction of the minor diameter of the stalk) with a stainless steel probe. The probe was 2 mm in diameter with a 45-degree 1 mm chamfer on its end (see Fig. 1). The probe was displaced at a constant rate of 25.4 mm/sec until it had completely punctured the stalk and reached a point 5 mm below the zero plane. The zero plane was defined as the bottom most part of the stalk being punctured. Figure 2 illustrates the test setup. Data acquisition was accomplished using Bluehill Universal testing software (Illinois ToolWorks Inc., Glenview IL). Both displacement and force were measured synchronously at a rate of 1000 samples per second.

**Fig. 3:**
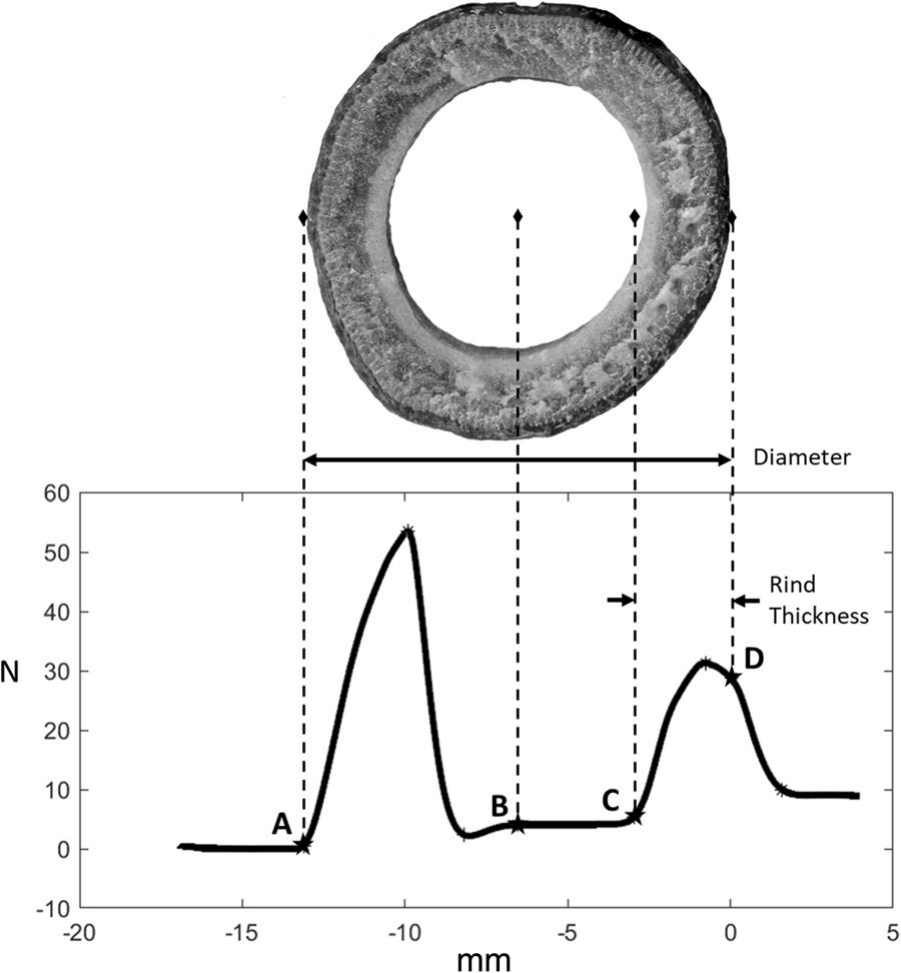
A demonstration of the key points of the load–extension curve and how each relates to the physical features of the stalk cross section. Labeled points are: A—Point of initial contact, B—Midpoint, C—Point of reengagement, D—Exit (zero) plane. Diameter is calculated as the distance between points A and D whereas the rind thickness is calculated as the distance between points C and D

A typical force displacement graph from a rind puncture test of a poison hemlock sample in shown in Fig. 3. A cross section of the same sample is shown above the force–displacement curve to illustrate the relationship between diameter, rind thickness and features of the force displacement graph. A custom automated MATLAB (Mathworks, Natick MA) algorithm (explained in further detail below) was developed to identify key points of the force–displacement curve generated during puncture testing and to calculate diameter and rind thickness.

The diameter was calculated by finding the distance from the point of initial contact (point A in Fig. 3) to the zero plane (point D in Fig. 3). The algorithm identified the point of initial contact using thresholding techniques to identify the first instance at which the force became nonzero. The first several data points were excluded from this analysis as non-zero forces occur when the probe is first put into motion due to inertial effects. After identifying the point of initial contact and the zero plane, the midpoint of the data was calculated (i.e., the center of the cross-section or point B in Fig. 3). The rind thickness of the stalk was determined by analyzing the data between the midpoint and the zero plane. In particular, the reengagement point (point C in Fig. 3) was identified using thresholding techniques on the second derivative of the force data to determine the point at which the force began to rapidly increase. This rapid increase in force was due to the tip of the probe reencountering the rind after traveling through the hollow center of the stalks cross-section. The rind thickness was defined as the distance between the reengagement point and the zero plane (see Additional files 1, 2, 3, 4 which contain sample test data as well as a copy of the Matlab algorithm and a copy of the Instron method file used during puncture testing).

### Caliper measurements of rind thickness

After rind puncture testing, each internode was cut in half immediately apical of the puncture location using a sharp straight edged knife. Calipers were then used to measure the rind thickness. Rind thickness measurements were acquired as near to the puncture location as possible (less than 25 mm from puncture location for all samples). Two researchers took independent measurements of rind thickness using the same set of digital calipers.

### Image analysis method of acquiring rind thickness and diameter

Each internode sample was then imaged and analyzed in ImageJ to determine the rind thickness and diameter. A small cross-sectional sample of the stalk was removed and scanned on an open bed scanner. If the sample broke apart or cracked while it was being sectioned, another attempt was made to cut an adjacent section. However, if the cross-sectional sample could not be made within 25 mm of the previous puncture and caliper measurements then no image data was collected on that internode.

Each cross-sectional sample was placed precisely on the open bed scanner to simplify identification of the location of previous caliper measurements of rind thickness and diameter. Scans were then acquired at 2400 dots per inch in full color. Images were imported into ImageJ to determine rind thickness and diameter. In particular, the ImageJ software was used to determine diameter and rind thickness by manually selecting points in the scanned image. The distance between the points was computed in units of pixels which were then converted to lengths in millimeters using a conversion factor based on the known pixel density of the scanner settings.

## Supplementary information

**Additional file 1: Instron test method file.** This file contains the control parameters for running a puncture test on an Instron universal testing system. The file can be used on any universal testing system which utilizes “Bluehill” software.

**Additional file 2: Sample puncture data.** These files are comma delimited csv files which contain force displacement data gathered from the instron universal testing system during puncture tests of poison hemlock.

**Additional file 3: Matlab algorithm for determining rind thickness and diameter**. This is a matlab file that is used to analyze force displacement data from puncture test and determine the samples rind thickness and diameter.

**Additional file 4: Graphs of Sample Data from Matlab Algorithm**. These .png files display the graphical output of matlab algorithm for each of the included sample puncture data files.

## Data Availability

The data sets obtained and analyzed during the current study are available from the corresponding author upon reasonable request.
