# Supplementary figures and images for: A novel rind puncture technique to measure rind thickness and diameter in plant stalks
Source: Plant Methods. 2020 Apr 1;16:44. doi: 10.1186/s13007-020-00587-4 (PMC7110687; doi:10.1186/s13007-020-00587-4)

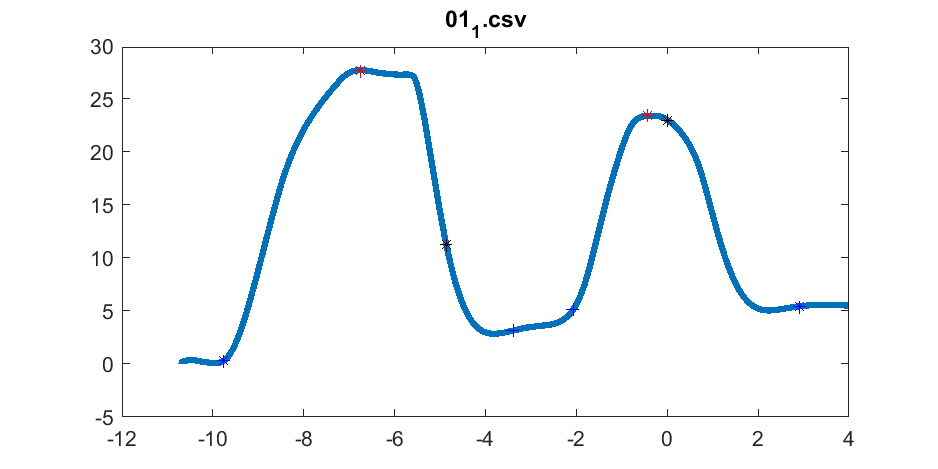

Supplement: Supplementary file 4 — Additional file 4: Graphs of Sample Data from Matlab Algorithm. These .png files display the graphical output of matlab algorithm for each of the included sample puncture data files. [file 13007_2020_587_MOESM4_ESM.zip › 01_1.csv.png]

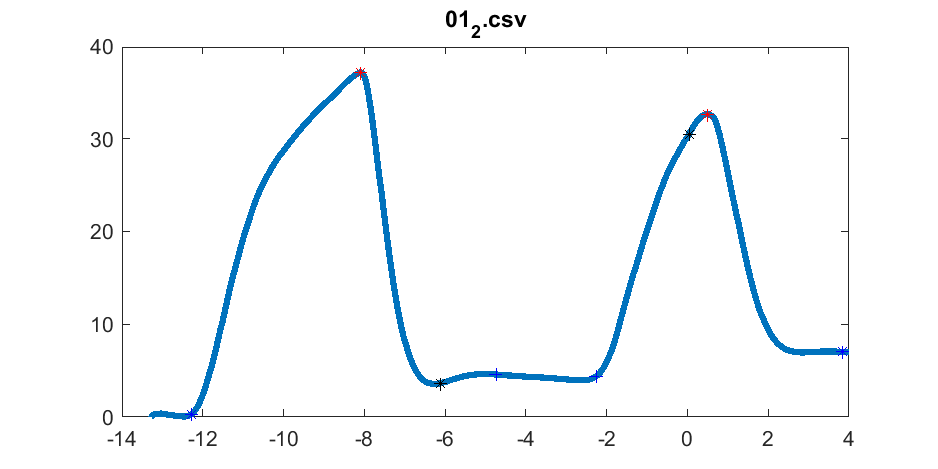

Supplement: Supplementary file 4 — Additional file 4: Graphs of Sample Data from Matlab Algorithm. These .png files display the graphical output of matlab algorithm for each of the included sample puncture data files. [file 13007_2020_587_MOESM4_ESM.zip › 01_2.csv.png]

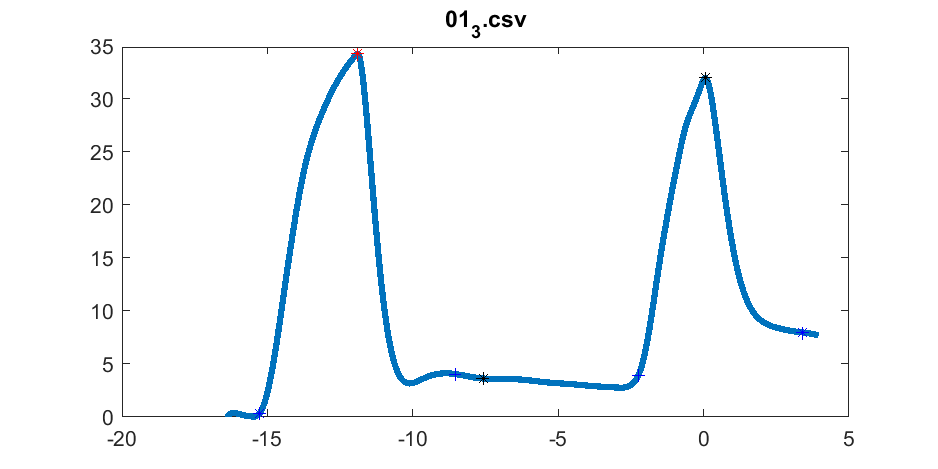

Supplement: Supplementary file 4 — Additional file 4: Graphs of Sample Data from Matlab Algorithm. These .png files display the graphical output of matlab algorithm for each of the included sample puncture data files. [file 13007_2020_587_MOESM4_ESM.zip › 01_3.csv.png]

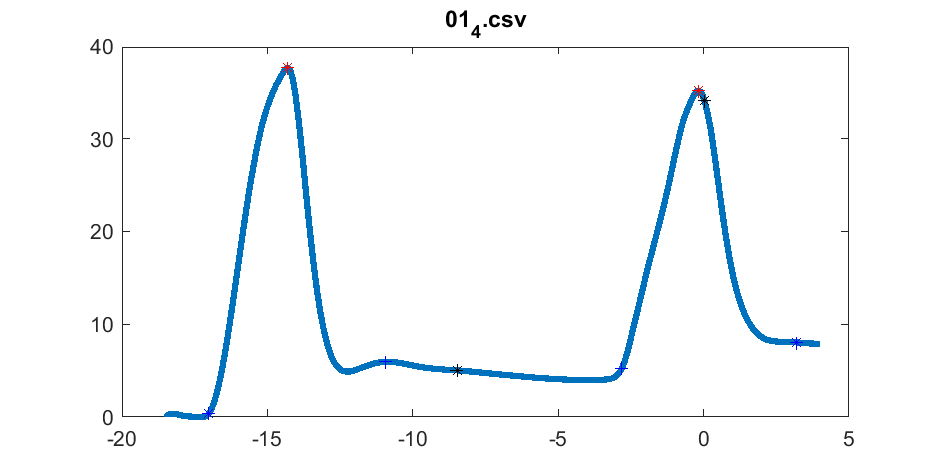

Supplement: Supplementary file 4 — Additional file 4: Graphs of Sample Data from Matlab Algorithm. These .png files display the graphical output of matlab algorithm for each of the included sample puncture data files. [file 13007_2020_587_MOESM4_ESM.zip › 01_4.csv.png]

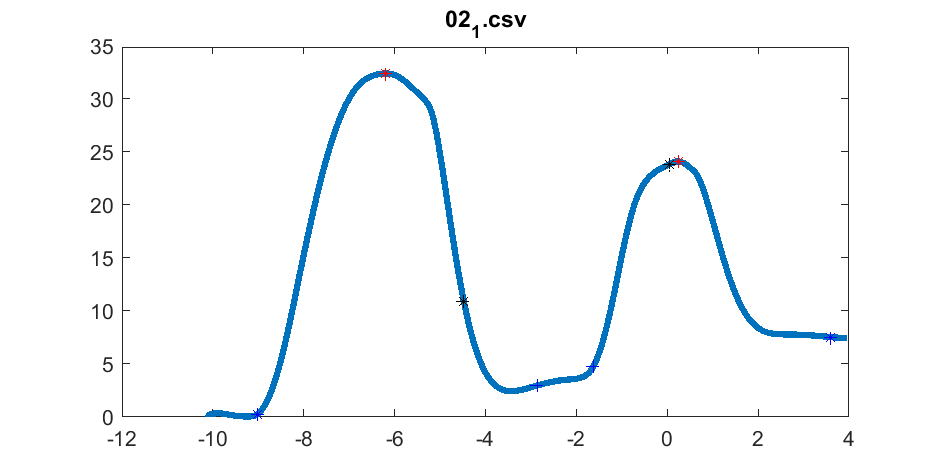

Supplement: Supplementary file 4 — Additional file 4: Graphs of Sample Data from Matlab Algorithm. These .png files display the graphical output of matlab algorithm for each of the included sample puncture data files. [file 13007_2020_587_MOESM4_ESM.zip › 02_1.csv.png]

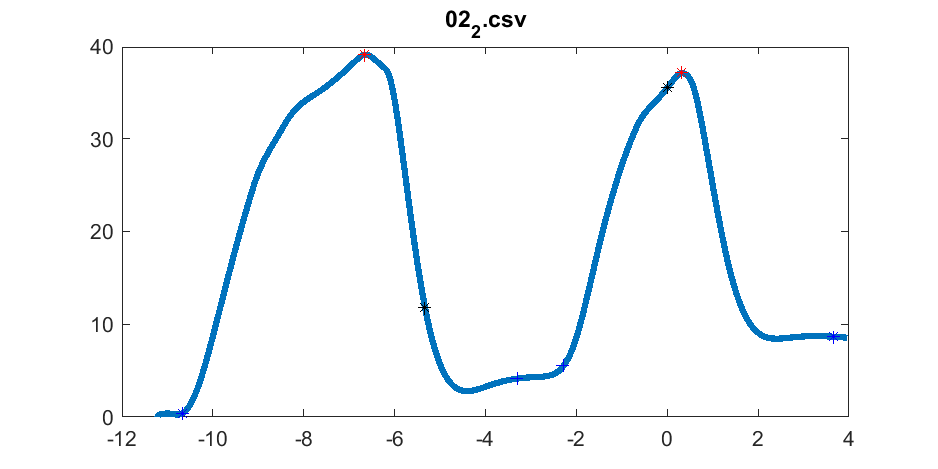

Supplement: Supplementary file 4 — Additional file 4: Graphs of Sample Data from Matlab Algorithm. These .png files display the graphical output of matlab algorithm for each of the included sample puncture data files. [file 13007_2020_587_MOESM4_ESM.zip › 02_2.csv.png]

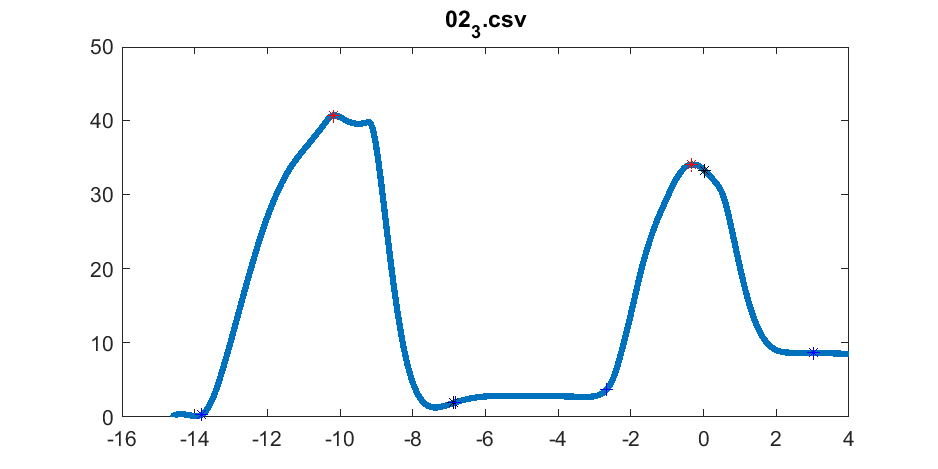

Supplement: Supplementary file 4 — Additional file 4: Graphs of Sample Data from Matlab Algorithm. These .png files display the graphical output of matlab algorithm for each of the included sample puncture data files. [file 13007_2020_587_MOESM4_ESM.zip › 02_3.csv.png]

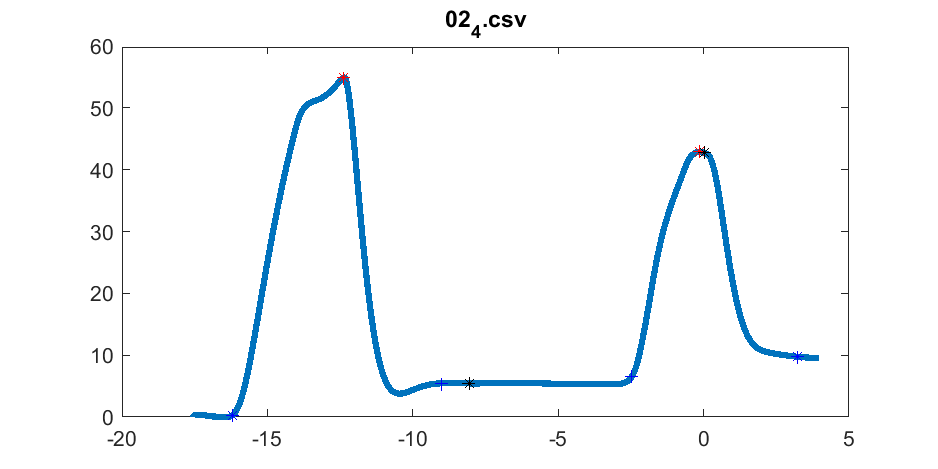

Supplement: Supplementary file 4 — Additional file 4: Graphs of Sample Data from Matlab Algorithm. These .png files display the graphical output of matlab algorithm for each of the included sample puncture data files. [file 13007_2020_587_MOESM4_ESM.zip › 02_4.csv.png]

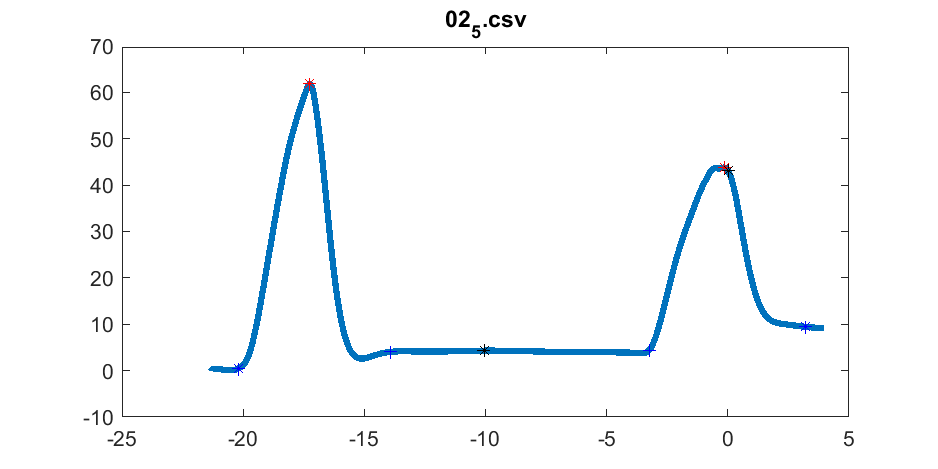

Supplement: Supplementary file 4 — Additional file 4: Graphs of Sample Data from Matlab Algorithm. These .png files display the graphical output of matlab algorithm for each of the included sample puncture data files. [file 13007_2020_587_MOESM4_ESM.zip › 02_5.csv.png]

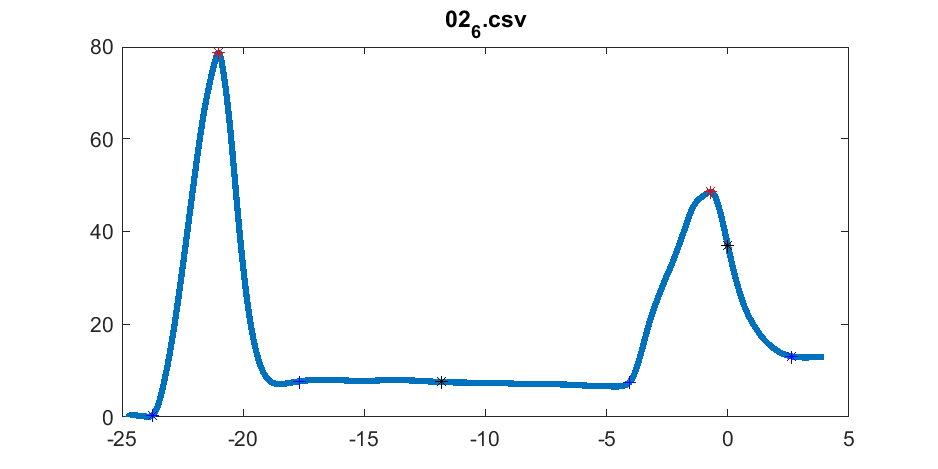

Supplement: Supplementary file 4 — Additional file 4: Graphs of Sample Data from Matlab Algorithm. These .png files display the graphical output of matlab algorithm for each of the included sample puncture data files. [file 13007_2020_587_MOESM4_ESM.zip › 02_6.csv.png]

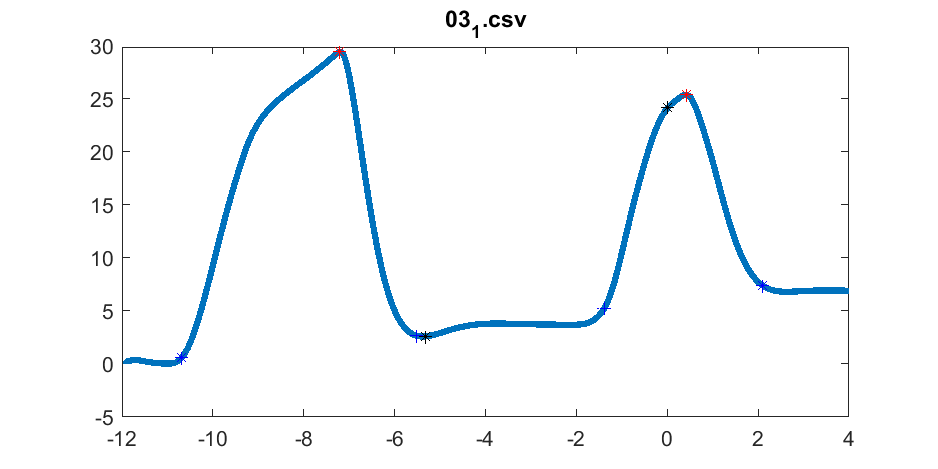

Supplement: Supplementary file 4 — Additional file 4: Graphs of Sample Data from Matlab Algorithm. These .png files display the graphical output of matlab algorithm for each of the included sample puncture data files. [file 13007_2020_587_MOESM4_ESM.zip › 03_1.csv.png]

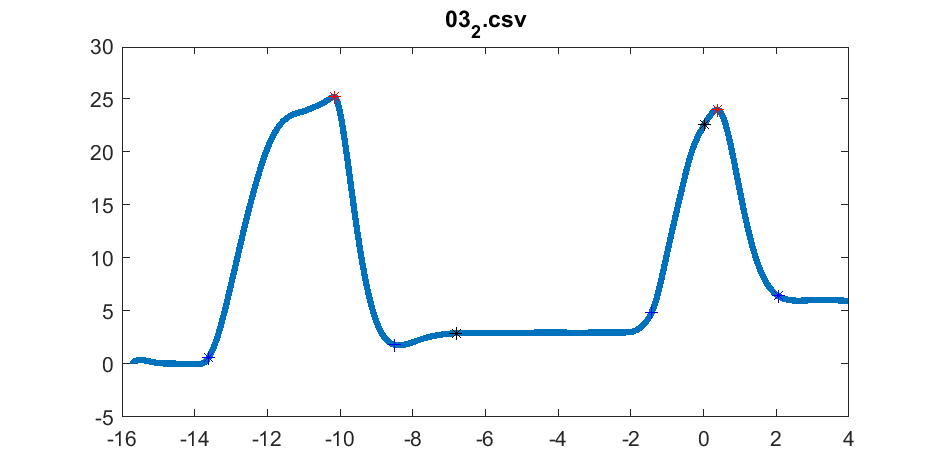

Supplement: Supplementary file 4 — Additional file 4: Graphs of Sample Data from Matlab Algorithm. These .png files display the graphical output of matlab algorithm for each of the included sample puncture data files. [file 13007_2020_587_MOESM4_ESM.zip › 03_2.csv.png]

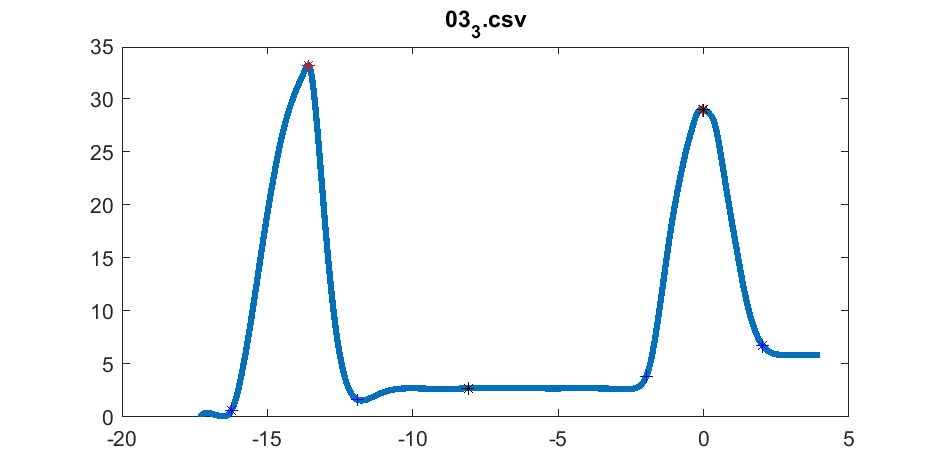

Supplement: Supplementary file 4 — Additional file 4: Graphs of Sample Data from Matlab Algorithm. These .png files display the graphical output of matlab algorithm for each of the included sample puncture data files. [file 13007_2020_587_MOESM4_ESM.zip › 03_3.csv.png]

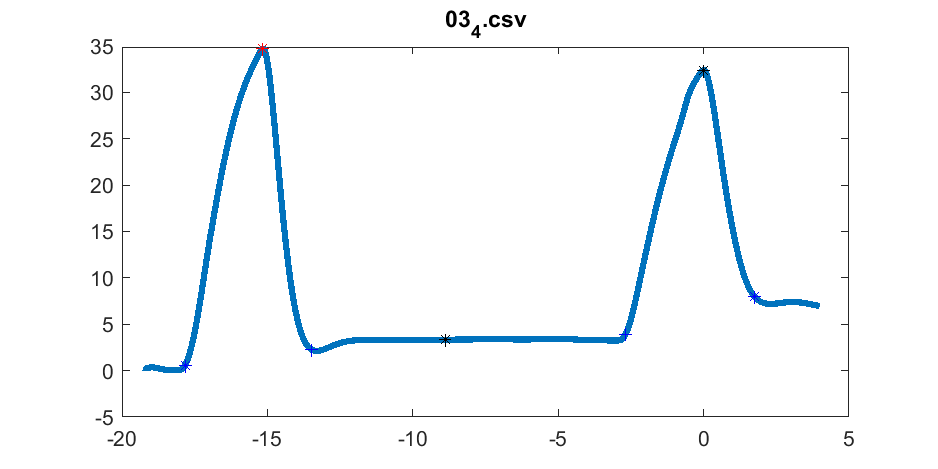

Supplement: Supplementary file 4 — Additional file 4: Graphs of Sample Data from Matlab Algorithm. These .png files display the graphical output of matlab algorithm for each of the included sample puncture data files. [file 13007_2020_587_MOESM4_ESM.zip › 03_4.csv.png]
